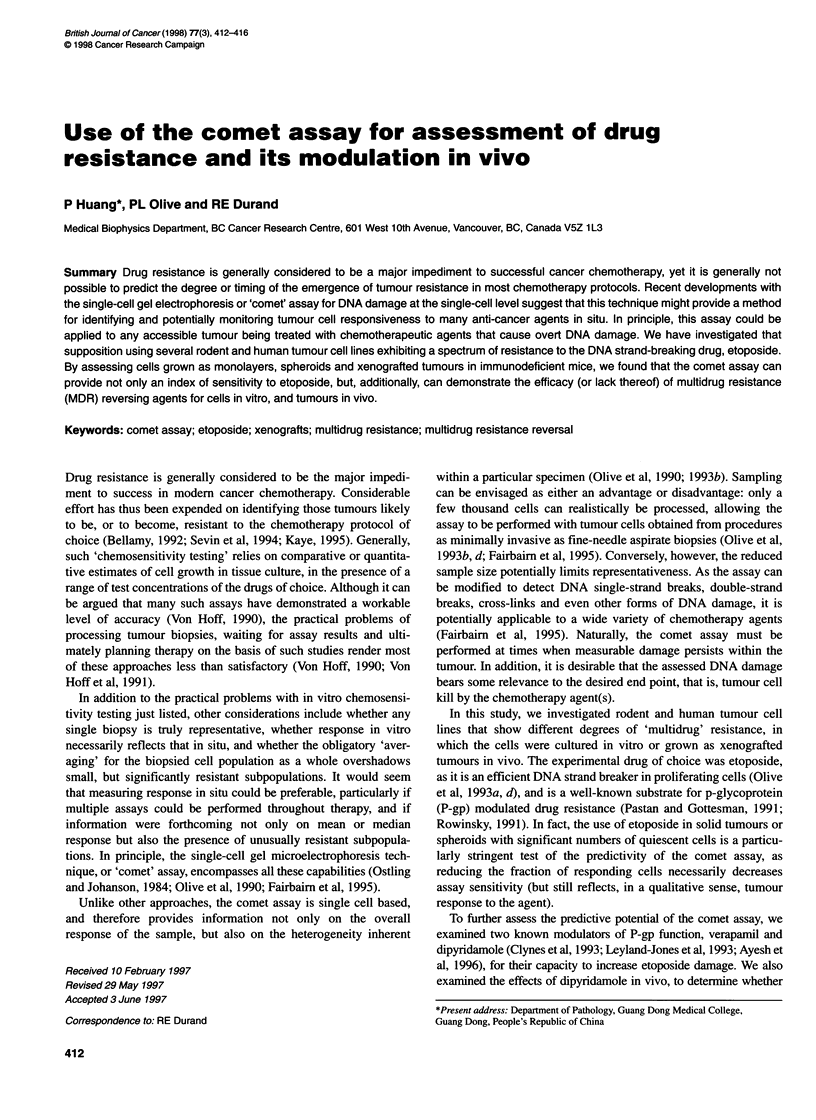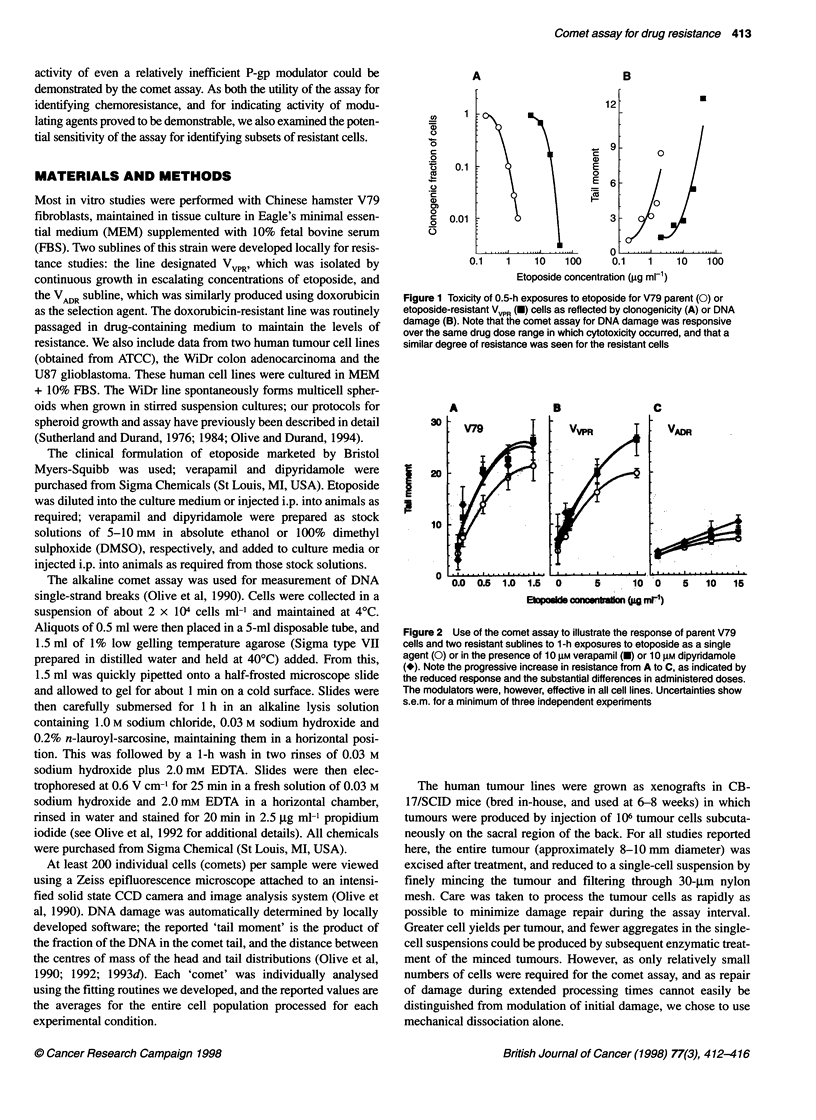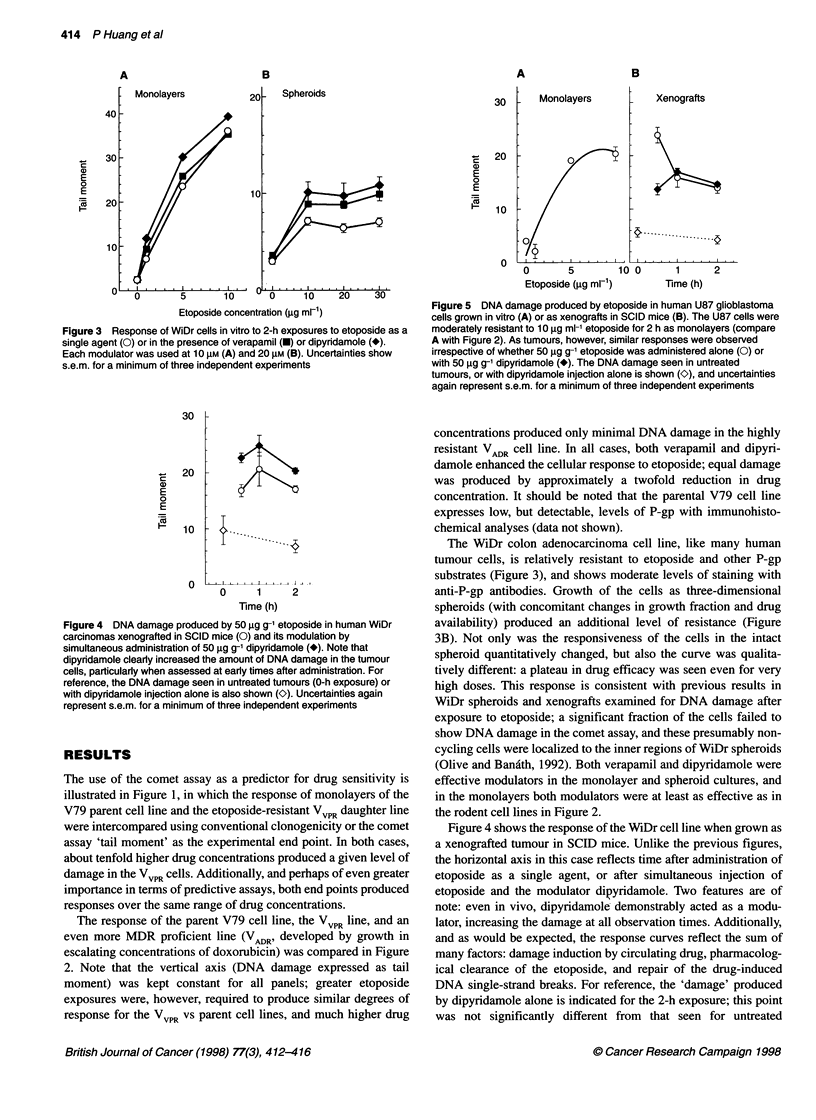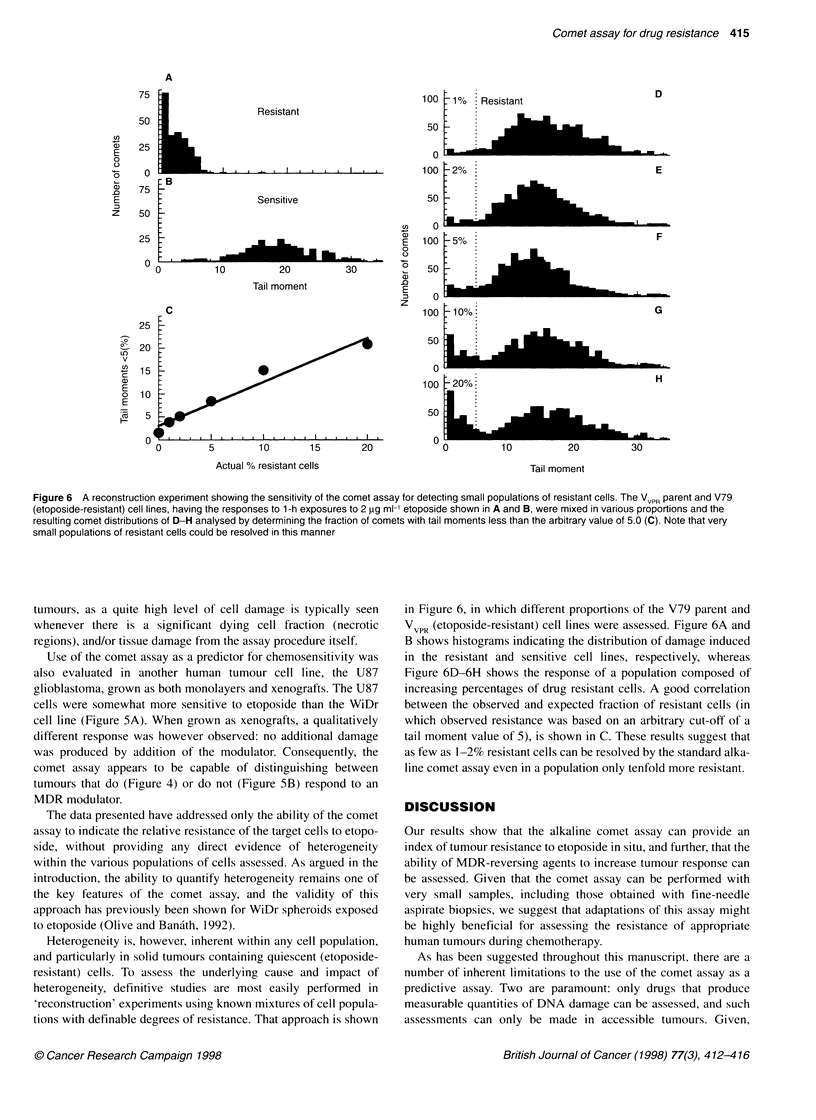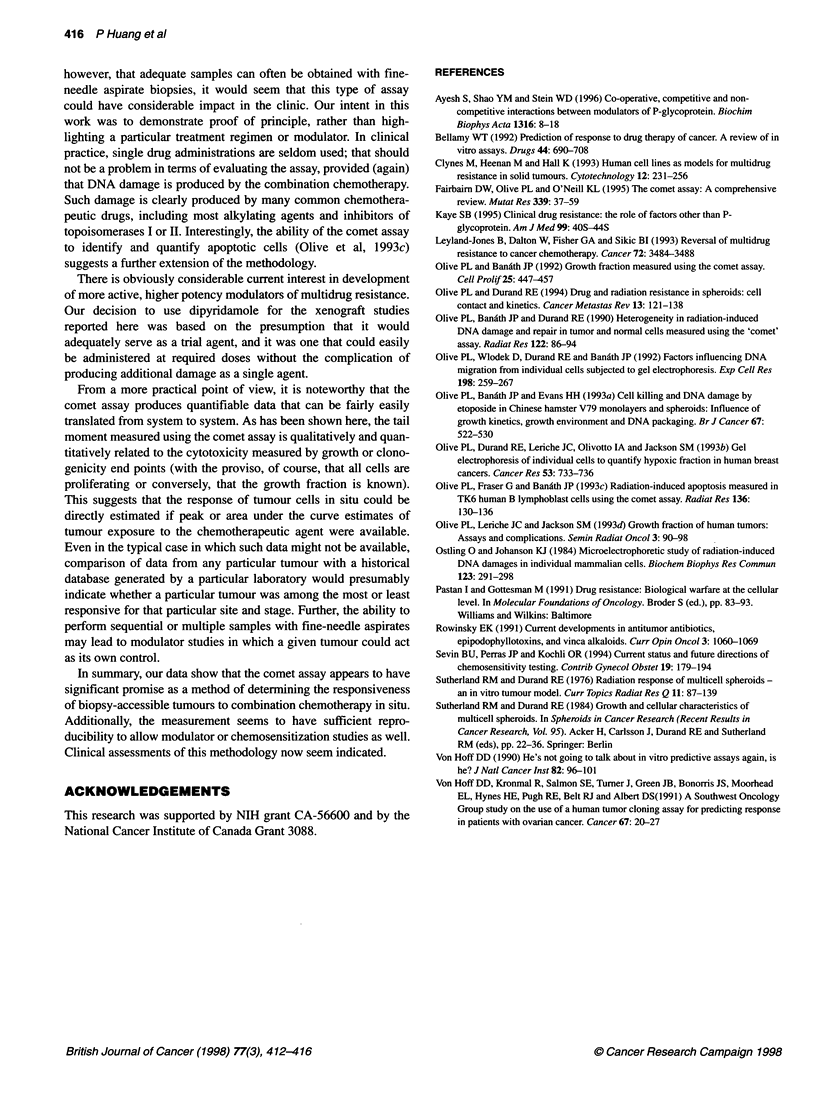# Use of the comet assay for assessment of drug resistance and its modulation in vivo.

**DOI:** 10.1038/bjc.1998.65

**Published:** 1998

**Authors:** P. Huang, P. L. Olive, R. E. Durand

**Affiliations:** Medical Biophysics Department, BC Cancer Research Centre, Vancouver, Canada.

## Abstract

Drug resistance is generally considered to be a major impediment to successful cancer chemotherapy, yet it is generally not possible to predict the degree or timing of the emergence of tumour resistance in most chemotherapy protocols. Recent developments with the single-cell gel electrophoresis or 'comet' assay for DNA damage at the single-cell level suggest that this technique might provide a method for identifying and potentially monitoring tumour cell responsiveness to many anti-cancer agents in situ. In principle, this assay could be applied to any accessible tumour being treated with chemotherapeutic agents that cause overt DNA damage. We have investigated that supposition using several rodent and human tumour cell lines exhibiting a spectrum of resistance to the DNA strand-breaking drug, etoposide. By assessing cells grown as monolayers, spheroids and xenografted tumours in immunodeficient mice, we found that the comet assay can provide not only an index of sensitivity to etoposide, but, additionally, can demonstrate the efficacy (or lack thereof) of multidrug resistance (MDR) reversing agents for cells in vitro, and tumours in vivo.


*British Journal of Cancer* (1998) **77**(3), 412–416
© 1998 Cancer Research Campaign

### Use of the comet assay for assessment of drug resistance and its modulation in vivo

**P Huang*, PL Olive and RE Durand**

Medical Biophysics Department, BC Cancer Research Centre, 601 West 10th Avenue, Vancouver, BC, Canada V5Z 1L3

**Summary** Drug resistance is generally considered to be a major impediment to successful cancer chemotherapy, yet it is generally not possible to predict the degree or timing of the emergence of tumour resistance in most chemotherapy protocols. Recent developments with the single-cell gel electrophoresis or 'comet' assay for DNA damage at the single-cell level suggest that this technique might provide a method for identifying and potentially monitoring tumour cell responsiveness to many anti-cancer agents in situ. In principle, this assay could be applied to any accessible tumour being treated with chemotherapeutic agents that cause overt DNA damage. We have investigated that supposition using several rodent and human tumour cell lines exhibiting a spectrum of resistance to the DNA strand-breaking drug, etoposide. By assessing cells grown as monolayers, spheroids and xenografted tumours in immunodeficient mice, we found that the comet assay can provide not only an index of sensitivity to etoposide, but, additionally, can demonstrate the efficacy (or lack thereof) of multidrug resistance (MDR) reversing agents for cells in vitro, and tumours in vivo.

**Keywords:** comet assay; etoposide; xenografts; multidrug resistance; multidrug resistance reversal

Drug resistance is generally considered to be the major impediment to success in modern cancer chemotherapy. Considerable effort has thus been expended on identifying those tumours likely to be, or to become, resistant to the chemotherapy protocol of choice (Bellamy, 1992; Sevin et al, 1994; Kaye, 1995). Generally, such 'chemosensitivity testing' relies on comparative or quantitative estimates of cell growth in tissue culture, in the presence of a range of test concentrations of the drugs of choice. Although it can be argued that many such assays have demonstrated a workable level of accuracy (Von Hoff, 1990), the practical problems of processing tumour biopsies, waiting for assay results and ultimately planning therapy on the basis of such studies render most of these approaches less than satisfactory (Von Hoff, 1990; Von Hoff et al, 1991).

In addition to the practical problems with in vitro chemosensitivity testing just listed, other considerations include whether any single biopsy is truly representative, whether response in vitro necessarily reflects that in situ, and whether the obligatory 'averaging' for the biopsied cell population as a whole overshadows small, but significantly resistant subpopulations. It would seem that measuring response in situ could be preferable, particularly if multiple assays could be performed throughout therapy, and if information were forthcoming not only on mean or median response but also the presence of unusually resistant subpopulations. In principle, the single-cell gel microelectrophoresis technique, or 'comet' assay, encompasses all these capabilities (Ostling and Johanson, 1984; Olive et al, 1990; Fairbairn et al, 1995).

Unlike other approaches, the comet assay is single cell based, and therefore provides information not only on the overall response of the sample, but also on the heterogeneity inherent within a particular specimen (Olive et al, 1990; 1993*b*). Sampling can be envisaged as either an advantage or disadvantage: only a few thousand cells can realistically be processed, allowing the assay to be performed with tumour cells obtained from procedures as minimally invasive as fine-needle aspirate biopsies (Olive et al, 1993*b*, *d*; Fairbairn et al, 1995). Conversely, however, the reduced sample size potentially limits representativeness. As the assay can be modified to detect DNA single-strand breaks, double-strand breaks, cross-links and even other forms of DNA damage, it is potentially applicable to a wide variety of chemotherapy agents (Fairbairn et al, 1995). Naturally, the comet assay must be performed at times when measurable damage persists within the tumour. In addition, it is desirable that the assessed DNA damage bears some relevance to the desired end point, that is, tumour cell kill by the chemotherapy agent(s).

In this study, we investigated rodent and human tumour cell lines that show different degrees of 'multidrug' resistance, in which the cells were cultured in vitro or grown as xenografted tumours in vivo. The experimental drug of choice was etoposide, as it is an efficient DNA strand breaker in proliferating cells (Olive et al, 1993*a*, *d*), and is a well-known substrate for p-glycoprotein (P-gp) modulated drug resistance (Pastan and Gottesman, 1991; Rowinsky, 1991). In fact, the use of etoposide in solid tumours or spheroids with significant numbers of quiescent cells is a particularly stringent test of the predictivity of the comet assay, as reducing the fraction of responding cells necessarily decreases assay sensitivity (but still reflects, in a qualitative sense, tumour response to the agent).

To further assess the predictive potential of the comet assay, we examined two known modulators of P-gp function, verapamil and dipyridamole (Clynes et al, 1993; Leyland-Jones et al, 1993; Ayesh et al, 1996), for their capacity to increase etoposide damage. We also examined the effects of dipyridamole in vivo, to determine whether

*Received 10 February 1997*
*Revised 29 May 1997*
*Accepted 3 June 1997*

*Correspondence to:* RE Durand

**Present address:* Department of Pathology, Guang Dong Medical College, Guang Dong, People's Republic of China activity of even a relatively inefficient P-gp modulator could be demonstrated by the comet assay. As both the utility of the assay for identifying chemoresistance, and for indicating activity of modulating agents proved to be demonstrable, we also examined the potential sensitivity of the assay for identifying subsets of resistant cells.

### MATERIALS AND METHODS

Most in vitro studies were performed with Chinese hamster V79 fibroblasts, maintained in tissue culture in Eagle's minimal essential medium (MEM) supplemented with 10% fetal bovine serum (FBS). Two sublines of this strain were developed locally for resistance studies: the line designated $V_{VPR}$, which was isolated by continuous growth in escalating concentrations of etoposide, and the $V_{ADR}$ subline, which was similarly produced using doxorubicin as the selection agent. The doxorubicin-resistant line was routinely passaged in drug-containing medium to maintain the levels of resistance. We also include data from two human tumour cell lines (obtained from ATCC), the WiDr colon adenocarcinoma and the U87 glioblastoma. These human cell lines were cultured in MEM + 10% FBS. The WiDr line spontaneously forms multicell spheroids when grown in stirred suspension cultures; our protocols for spheroid growth and assay have previously been described in detail (Sutherland and Durand, 1976; 1984; Olive and Durand, 1994).

The clinical formulation of etoposide marketed by Bristol Myers-Squibb was used; verapamil and dipyridamole were purchased from Sigma Chemicals (St Louis, MI, USA). Etoposide was diluted into the culture medium or injected i.p. into animals as required; verapamil and dipyridamole were prepared as stock solutions of 5–10 mM in absolute ethanol or 100% dimethyl sulphoxide (DMSO), respectively, and added to culture media or injected i.p. into animals as required from those stock solutions.

The alkaline comet assay was used for measurement of DNA single-strand breaks (Olive et al, 1990). Cells were collected in a suspension of about $2 \times 10^4$ cells $ml^{-1}$ and maintained at 4°C. Aliquots of 0.5 ml were then placed in a 5-ml disposable tube, and 1.5 ml of 1% low gelling temperature agarose (Sigma type VII prepared in distilled water and held at 40°C) added. From this, 1.5 ml was quickly pipetted onto a half-frosted microscope slide and allowed to gel for about 1 min on a cold surface. Slides were then carefully submersed for 1 h in an alkaline lysis solution containing 1.0 M sodium chloride, 0.03 M sodium hydroxide and 0.2% *n*-lauroyl-sarcosine, maintaining them in a horizontal position. This was followed by a 1-h wash in two rinses of 0.03 M sodium hydroxide plus 2.0 mM EDTA. Slides were then electrophoresed at 0.6 V $cm^{-1}$ for 25 min in a fresh solution of 0.03 M sodium hydroxide and 2.0 mM EDTA in a horizontal chamber, rinsed in water and stained for 20 min in 2.5 μg $ml^{-1}$ propidium iodide (see Olive et al, 1992 for additional details). All chemicals were purchased from Sigma Chemical (St Louis, MI, USA).

At least 200 individual cells (comets) per sample were viewed using a Zeiss epifluorescence microscope attached to an intensified solid state CCD camera and image analysis system (Olive et al, 1990). DNA damage was automatically determined by locally developed software; the reported 'tail moment' is the product of the fraction of the DNA in the comet tail, and the distance between the centres of mass of the head and tail distributions (Olive et al, 1990; 1992; 1993*d*). Each 'comet' was individually analysed using the fitting routines we developed, and the reported values are the averages for the entire cell population processed for each experimental condition.

**Figure 1** Toxicity of 0.5-h exposures to etoposide for V79 parent (○) or etoposide-resistant $V_{VPR}$ (■) cells as reflected by clonogenicity (**A**) or DNA damage (**B**). Note that the comet assay for DNA damage was responsive over the same drug dose range in which cytotoxicity occurred, and that a similar degree of resistance was seen for the resistant cells

**Figure 2** Use of the comet assay to illustrate the response of parent V79 cells and two resistant sublines to 1-h exposures to etoposide as a single agent (○) or in the presence of 10 μM verapamil (■) or 10 μM dipyridamole (◆). Note the progressive increase in resistance from **A** to **C**, as indicated by the reduced response and the substantial differences in administered doses. The modulators were, however, effective in all cell lines. Uncertainties show s.e.m. for a minimum of three independent experiments

The human tumour lines were grown as xenografts in CB-17/SCID mice (bred in-house, and used at 6–8 weeks) in which tumours were produced by injection of $10^6$ tumour cells subcutaneously on the sacral region of the back. For all studies reported here, the entire tumour (approximately 8–10 mm diameter) was excised after treatment, and reduced to a single-cell suspension by finely mincing the tumour and filtering through 30-μm nylon mesh. Care was taken to process the tumour cells as rapidly as possible to minimize damage repair during the assay interval. Greater cell yields per tumour, and fewer aggregates in the single-cell suspensions could be produced by subsequent enzymatic treatment of the minced tumours. However, as only relatively small numbers of cells were required for the comet assay, and as repair of damage during extended processing times cannot easily be distinguished from modulation of initial damage, we chose to use mechanical dissociation alone.

*© Cancer Research Campaign 1998* 

**Figure 3** Response of WiDr cells in vitro to 2-h exposures to etoposide as a single agent (○) or in the presence of verapamil (■) or dipyridamole (◆). Each modulator was used at 10 μM (**A**) and 20 μM (**B**). Uncertainties show s.e.m. for a minimum of three independent experiments

**Figure 5** DNA damage produced by etoposide in human U87 glioblastoma cells grown in vitro (**A**) or as xenografts in SCID mice (**B**). The U87 cells were moderately resistant to 10 μg ml⁻¹ etoposide for 2 h as monolayers (compare **A** with Figure 2). As tumours, however, similar responses were observed irrespective of whether 50 μg g⁻¹ etoposide was administered alone (○) or with 50 μg g⁻¹ dipyridamole (◆). The DNA damage seen in untreated tumours, or with dipyridamole injection alone is shown (◇), and uncertainties again represent s.e.m. for a minimum of three independent experiments

**Figure 4** DNA damage produced by 50 μg g⁻¹ etoposide in human WiDr carcinomas xenografted in SCID mice (○) and its modulation by simultaneous administration of 50 μg g⁻¹ dipyridamole (◆). Note that dipyridamole clearly increased the amount of DNA damage in the tumour cells, particularly when assessed at early times after administration. For reference, the DNA damage seen in untreated tumours (0-h exposure) or with dipyridamole injection alone is also shown (◇). Uncertainties again represent s.e.m. for a minimum of three independent experiments

### RESULTS

The use of the comet assay as a predictor for drug sensitivity is illustrated in Figure 1, in which the response of monolayers of the V79 parent cell line and the etoposide-resistant $V_{VPR}$ daughter line were intercompared using conventional clonogenicity or the comet assay 'tail moment' as the experimental end point. In both cases, about tenfold higher drug concentrations produced a given level of damage in the $V_{VPR}$ cells. Additionally, and perhaps of even greater importance in terms of predictive assays, both end points produced responses over the same range of drug concentrations.

The response of the parent V79 cell line, the $V_{VPR}$ line, and an even more MDR proficient line ($V_{ADR}$, developed by growth in escalating concentrations of doxorubicin) was compared in Figure 2. Note that the vertical axis (DNA damage expressed as tail moment) was kept constant for all panels; greater etoposide exposures were, however, required to produce similar degrees of response for the $V_{VPR}$ vs parent cell lines, and much higher drug concentrations produced only minimal DNA damage in the highly resistant $V_{ADR}$ cell line. In all cases, both verapamil and dipyridamole enhanced the cellular response to etoposide; equal damage was produced by approximately a twofold reduction in drug concentration. It should be noted that the parental V79 cell line expresses low, but detectable, levels of P-gp with immunohistochemical analyses (data not shown).

The WiDr colon adenocarcinoma cell line, like many human tumour cells, is relatively resistant to etoposide and other P-gp substrates (Figure 3), and shows moderate levels of staining with anti-P-gp antibodies. Growth of the cells as three-dimensional spheroids (with concomitant changes in growth fraction and drug availability) produced an additional level of resistance (Figure 3B). Not only was the responsiveness of the cells in the intact spheroid quantitatively changed, but also the curve was qualitatively different: a plateau in drug efficacy was seen even for very high doses. This response is consistent with previous results in WiDr spheroids and xenografts examined for DNA damage after exposure to etoposide; a significant fraction of the cells failed to show DNA damage in the comet assay, and these presumably non-cycling cells were localized to the inner regions of WiDr spheroids (Olive and Banáth, 1992). Both verapamil and dipyridamole were effective modulators in the monolayer and spheroid cultures, and in the monolayers both modulators were at least as effective as in the rodent cell lines in Figure 2.

Figure 4 shows the response of the WiDr cell line when grown as a xenografted tumour in SCID mice. Unlike the previous figures, the horizontal axis in this case reflects time after administration of etoposide as a single agent, or after simultaneous injection of etoposide and the modulator dipyridamole. Two features are of note: even in vivo, dipyridamole demonstrably acted as a modulator, increasing the damage at all observation times. Additionally, and as would be expected, the response curves reflect the sum of many factors: damage induction by circulating drug, pharmacological clearance of the etoposide, and repair of the drug-induced DNA single-strand breaks. For reference, the 'damage' produced by dipyridamole alone is indicated for the 2-h exposure; this point was not significantly different from that seen for untreated

© Cancer Research Campaign 1998

**Figure 6** A reconstruction experiment showing the sensitivity of the comet assay for detecting small populations of resistant cells. The $V_{VPR}$ parent and V79 (etoposide-resistant) cell lines, having the responses to 1-h exposures to 2 μg $ml^{-1}$ etoposide shown in **A** and **B**, were mixed in various proportions and the resulting comet distributions of **D**–**H** analysed by determining the fraction of comets with tail moments less than the arbitrary value of 5.0 (**C**). Note that very small populations of resistant cells could be resolved in this manner tumours, as a quite high level of cell damage is typically seen whenever there is a significant dying cell fraction (necrotic regions), and/or tissue damage from the assay procedure itself.

Use of the comet assay as a predictor for chemosensitivity was also evaluated in another human tumour cell line, the U87 glioblastoma, grown as both monolayers and xenografts. The U87 cells were somewhat more sensitive to etoposide than the WiDr cell line (Figure 5A). When grown as xenografts, a qualitatively different response was however observed: no additional damage was produced by addition of the modulator. Consequently, the comet assay appears to be capable of distinguishing between tumours that do (Figure 4) or do not (Figure 5B) respond to an MDR modulator.

The data presented have addressed only the ability of the comet assay to indicate the relative resistance of the target cells to etoposide, without providing any direct evidence of heterogeneity within the various populations of cells assessed. As argued in the introduction, the ability to quantify heterogeneity remains one of the key features of the comet assay, and the validity of this approach has previously been shown for WiDr spheroids exposed to etoposide (Olive and Banáth, 1992).

Heterogeneity is, however, inherent within any cell population, and particularly in solid tumours containing quiescent (etoposide-resistant) cells. To assess the underlying cause and impact of heterogeneity, definitive studies are most easily performed in 'reconstruction' experiments using known mixtures of cell populations with definable degrees of resistance. That approach is shown in Figure 6, in which different proportions of the V79 parent and $V_{VPR}$ (etoposide-resistant) cell lines were assessed. Figure 6A and B shows histograms indicating the distribution of damage induced in the resistant and sensitive cell lines, respectively, whereas Figure 6D–6H shows the response of a population composed of increasing percentages of drug resistant cells. A good correlation between the observed and expected fraction of resistant cells (in which observed resistance was based on an arbitrary cut-off of a tail moment value of 5), is shown in C. These results suggest that as few as 1–2% resistant cells can be resolved by the standard alkaline comet assay even in a population only tenfold more resistant.

### DISCUSSION

Our results show that the alkaline comet assay can provide an index of tumour resistance to etoposide in situ, and further, that the ability of MDR-reversing agents to increase tumour response can be assessed. Given that the comet assay can be performed with very small samples, including those obtained with fine-needle aspirate biopsies, we suggest that adaptations of this assay might be highly beneficial for assessing the resistance of appropriate human tumours during chemotherapy.

As has been suggested throughout this manuscript, there are a number of inherent limitations to the use of the comet assay as a predictive assay. Two are paramount: only drugs that produce measurable quantities of DNA damage can be assessed, and such assessments can only be made in accessible tumours. Given,

© Cancer Research Campaign 1998

however, that adequate samples can often be obtained with fine-needle aspirate biopsies, it would seem that this type of assay could have considerable impact in the clinic. Our intent in this work was to demonstrate proof of principle, rather than highlighting a particular treatment regimen or modulator. In clinical practice, single drug administrations are seldom used; that should not be a problem in terms of evaluating the assay, provided (again) that DNA damage is produced by the combination chemotherapy. Such damage is clearly produced by many common chemotherapeutic drugs, including most alkylating agents and inhibitors of topoisomerases I or II. Interestingly, the ability of the comet assay to identify and quantify apoptotic cells (Olive et al, 1993*c*) suggests a further extension of the methodology.

There is obviously considerable current interest in development of more active, higher potency modulators of multidrug resistance. Our decision to use dipyridamole for the xenograft studies reported here was based on the presumption that it would adequately serve as a trial agent, and it was one that could easily be administered at required doses without the complication of producing additional damage as a single agent.

From a more practical point of view, it is noteworthy that the comet assay produces quantifiable data that can be fairly easily translated from system to system. As has been shown here, the tail moment measured using the comet assay is qualitatively and quantitatively related to the cytotoxicity measured by growth or clonogenicity end points (with the proviso, of course, that all cells are proliferating or conversely, that the growth fraction is known). This suggests that the response of tumour cells in situ could be directly estimated if peak or area under the curve estimates of tumour exposure to the chemotherapeutic agent were available. Even in the typical case in which such data might not be available, comparison of data from any particular tumour with a historical database generated by a particular laboratory would presumably indicate whether a particular tumour was among the most or least responsive for that particular site and stage. Further, the ability to perform sequential or multiple samples with fine-needle aspirates may lead to modulator studies in which a given tumour could act as its own control.

In summary, our data show that the comet assay appears to have significant promise as a method of determining the responsiveness of biopsy-accessible tumours to combination chemotherapy in situ. Additionally, the measurement seems to have sufficient reproducibility to allow modulator or chemosensitization studies as well. Clinical assessments of this methodology now seem indicated.

### ACKNOWLEDGEMENTS

This research was supported by NIH grant CA-56600 and by the National Cancer Institute of Canada Grant 3088.

### REFERENCES

Ayesh S, Shao YM and Stein WD (1996) Co-operative, competitive and non-competitive interactions between modulators of P-glycoprotein. *Biochim Biophys Acta* **1316**: 8–18

Bellamy WT (1992) Prediction of response to drug therapy of cancer. A review of in vitro assays. *Drugs* **44**: 690–708

Clynes M, Heenan M and Hall K (1993) Human cell lines as models for multidrug resistance in solid tumours. *Cytotechnology* **12**: 231–256

Fairbairn DW, Olive PL and O'Neill KL (1995) The comet assay: A comprehensive review. *Mutat Res* **339**: 37–59

Kaye SB (1995) Clinical drug resistance: the role of factors other than P-glycoprotein. *Am J Med* **99**: 40S–44S

Leyland-Jones B, Dalton W, Fisher GA and Sikic BI (1993) Reversal of multidrug resistance to cancer chemotherapy. *Cancer* **72**: 3484–3488

Olive PL and Banáth JP (1992) Growth fraction measured using the comet assay. *Cell Prolif* **25**: 447–457

Olive PL and Durand RE (1994) Drug and radiation resistance in spheroids: cell contact and kinetics. *Cancer Metastas Rev* **13**: 121–138

Olive PL, Banáth JP and Durand RE (1990) Heterogeneity in radiation-induced DNA damage and repair in tumor and normal cells measured using the 'comet' assay. *Radiat Res* **122**: 86–94

Olive PL, Wlodek D, Durand RE and Banáth JP (1992) Factors influencing DNA migration from individual cells subjected to gel electrophoresis. *Exp Cell Res* **198**: 259–267

Olive PL, Banáth JP and Evans HH (1993*a*) Cell killing and DNA damage by etoposide in Chinese hamster V79 monolayers and spheroids: Influence of growth kinetics, growth environment and DNA packaging. *Br J Cancer* **67**: 522–530

Olive PL, Durand RE, Leriche JC, Olivotto IA and Jackson SM (1993*b*) Gel electrophoresis of individual cells to quantify hypoxic fraction in human breast cancers. *Cancer Res* **53**: 733–736

Olive PL, Fraser G and Banáth JP (1993*c*) Radiation-induced apoptosis measured in TK6 human B lymphoblast cells using the comet assay. *Radiat Res* **136**: 130–136

Olive PL, Leriche JC and Jackson SM (1993*d*) Growth fraction of human tumors: Assays and complications. *Semin Radiat Oncol* **3**: 90–98

Ostling O and Johanson KJ (1984) Microelectrophoretic study of radiation-induced DNA damages in individual mammalian cells. *Biochem Biophys Res Commun* **123**: 291–298

Pastan I and Gottesman M (1991) Drug resistance: Biological warfare at the cellular level. In *Molecular Foundations of Oncology*. Broder S (ed.), pp. 83–93. Williams and Wilkins: Baltimore

Rowinsky EK (1991) Current developments in antitumor antibiotics, epipodophyllotoxins, and vinca alkaloids. *Curr Opin Oncol* **3**: 1060–1069

Sevin BU, Perras JP and Kochli OR (1994) Current status and future directions of chemosensitivity testing. *Contrib Gynecol Obstet* **19**: 179–194

Sutherland RM and Durand RE (1976) Radiation response of multicell spheroids – an in vitro tumour model. *Curr Topics Radiat Res Q* **11**: 87–139

Sutherland RM and Durand RE (1984) Growth and cellular characteristics of multicell spheroids. In *Spheroids in Cancer Research (Recent Results in Cancer Research, Vol. 95)*. Acker H, Carlsson J, Durand RE and Sutherland RM (eds), pp. 22–36. Springer: Berlin

Von Hoff DD (1990) He's not going to talk about in vitro predictive assays again, is he? *J Natl Cancer Inst* **82**: 96–101

Von Hoff DD, Kronmal R, Salmon SE, Turner J, Green JB, Bonorris JS, Moorhead EL, Hynes HE, Pugh RE, Belt RJ and Albert DS(1991) A Southwest Oncology Group study on the use of a human tumor cloning assay for predicting response in patients with ovarian cancer. *Cancer* **67**: 20–27

© Cancer Research Campaign 1998